# Malignant neoplasms of the oral cavity and oropharynx treated in Brazil: what do hospital cancer records reveal?

**DOI:** 10.1016/j.bjorl.2020.05.019

**Published:** 2020-06-24

**Authors:** Sheilla de Oliveira Faria, Murilo César do Nascimento, Marco Aurélio Vamondes Kulcsar

**Affiliations:** a Universidade de São Paulo, Faculdade de Medicina (FMUSP), Departamento de Medicina Preventiva, São Paulo, SP, Brazil; b Universidade Federal de Alfenas (Unifal), Escola de Enfermagem, Alfenas, MG, Brazil; c Universidade de São Paulo, Faculdade de Medicina, Hospital das Clínicas (HCFMUSP), Instituto do Câncer do Estado de São Paulo, São Paulo, SP, Brazil

**Keywords:** Epidemiology, Head and neck neoplasms, Health services research, Cancer institutes, Database

## Abstract

**Introduction:**

Head and neck cancer has an impact on the global burden of diseases, representing an important cause of morbidity and mortality in Brazil, as well as worldwide.

**Objective:**

To learn and describe the clinical, epidemiological and care configuration provided to patients with cancer of the oral cavity and oropharynx recorded in Brazil, diagnosed from 2007 to 2016.

**Methods:**

This is a cross-sectional study, carried out using secondary hospital-based data, using the indirect documentation technique.

**Results:**

There were 52,799 hospital records of oral cavity cancer and 34,516 cases of oropharyngeal cancer in the assessed period. There was a predominance of male patients, aged 50–59 years, mostly Caucasians, and with a low level of schooling. Throughout the period there was a significant reduction in the positive history of alcohol and tobacco consumption, except for alcoholic beverages in oral cavity cancer. Most patients were diagnosed at an advanced stage of the disease (III or IV). Most patients with oral cavity cancer had no evidence of the disease on follow-up, while most patients with oropharyngeal cancer died. The first most frequent treatment offered to patients with oral cavity cancer was surgery, while for patients with oropharyngeal cancer it chemoradiotherapy predominated.

**Conclusion:**

Despite the fact that, in general, there was a reduction in the records of patient alcohol and tobacco consumption, the increase in the number of medical consultations, the predominantly late diagnosis and the patients’ low level of schooling indicate the need for health education, primary prevention and early diagnosis of cancer of the oral cavity and oropharynx.

## Introduction

According to data from GLOBOCAN, it is estimated that more than 1.2 million new cases of head and neck cancer will be diagnosed and about 680,000 deaths will be reported worldwide in 2040.^1^ In Brazil, according to an estimate by the National Cancer Institute (INCA), for each year of the 2020–2022 triennium, 22,840 new cases of cancer of the oral cavity, lip, oropharynx and larynx are expected.^2^ It is estimated that, of all head and neck cancers, cancers of the mouth and oropharynx are the most frequent ones.^1^, ^2^

The epidemiology of Head and Neck Cancer (HNC) has changed in recent years, as smoking-related HNCs have decreased in incidence, while cases of Human Papillomavirus (HPV)-related cancer have increased. In any case, smoking and alcoholism are still considered to be largely responsible for the high rate of HNC cases.^3^, ^4^ Other factors associated with HNC such as diet (poor in fruits and vegetables), poor oral hygiene and the role of the oral microbiome (especially of *Fusobacterium nucleatum* and *Porphyromonas gingivalis*) have been investigated.^5^, ^6^, ^7^

The standard treatment for HNC is based primarily on anatomical considerations and TNM staging (Tumor, Lymph Nodes, Metastasis).^8^ About 66% of cases are diagnosed at advanced stages (III or IV) and, therefore, imply more aggressive, expensive treatments, with a negative impact on both the quality of life and survival when compared to the early stages.^9^

In Brazil, national data on the notification of malignant neoplasms are centralized in the RHC (Hospital-Based Cancer Registry) Integrator, which joins approximately 25 information sites with data from 260 Brazilian hospitals, aiming to contribute to the improvement of patient care and intra-institutional planning.^10^

Knowledge of the clinical, epidemiological and care configuration of oral cavity and oropharyngeal cancers is essential for understanding their etiological and care aspects, as well as for proposing public health actions that can contribute to early detection and prevention. In view of the above, the aim of the present study was to identify the clinical-epidemiological and care profile of patients with oral cavity and oropharynx cancer treated in Brazil in the last decade.

## Methods

This is a cross-sectional study, carried out using secondary, hospital-based data. The data of the patients diagnosed between the years 2007 and 2016, registered in the database of the Hospital-based Cancer Registry System (SISRHC),^11^ were consulted in a timely manner, using the indirect documentation technique.

Hospital records of patients with the following head and neck malignant neoplasms were included, according to the International Classification of Diseases for Oncology (ICD-O), third edition (WHO, 2005)^12^: Oral Cavity (C00 – Lip; C02 – Other non-specific parts of the tongue; C03 – Gum; C04 – Floor of the mouth; C05 – Palate and C06 – Other non-specific parts of the mouth) and Oropharynx (C01 – Base of the tongue; C09 – Palatine Tonsils and C10 – Oropharynx). The data were obtained in November 2019.

The data on the medical consultations related to new cases of cancer of the oral cavity and oropharynx diagnosed per year were explored, stratified and presented as frequencies of the following variables: demographic (gender, age group), clinical/epidemiological/care data (staging, first treatment received in the hospital, disease stage at the end of treatment and smoking and/or alcoholism).

The data were exported to the Microsoft Excel program and analyzed using the Stata software (v. 13). The linear regression model was used for the trend analysis. For all analyses, 95% confidence interval was considered, with statistical significance set at *p* <  0.05.

Considering the guidelines and regulatory norms for research involving human beings, which is addressed in Resolution N. 466, of December 12, 2012,^13^ it is stated that, since this is a study of secondary data under public domain obtained from an Information System available for free access, it was not necessary to submit a proposal to the Research Ethics Committee.

## Results

**Figure 1:**
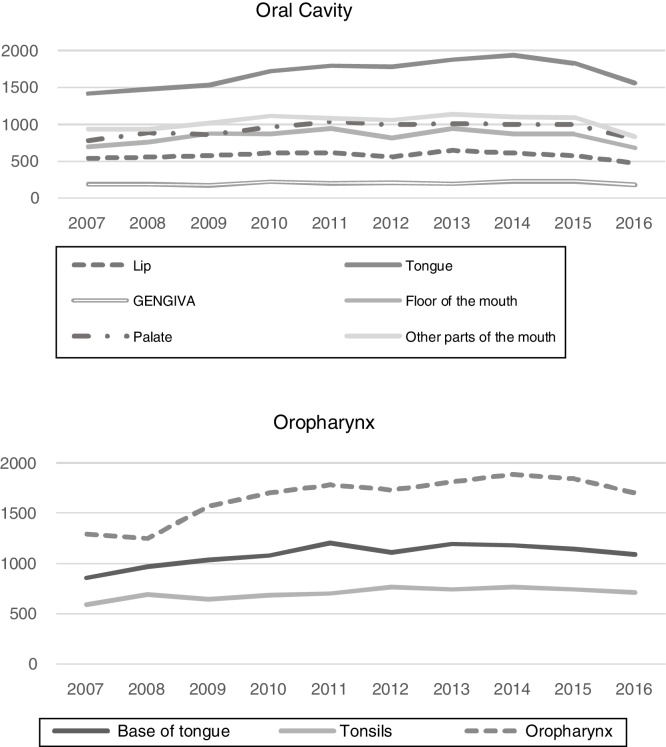
Numbers of new cases of oral cavity and oropharynx cancer recorded in Brazil, 2007‒2016.

Hospital records of 52,799 cases of oral cavity cancer and 34,516 cases of oropharyngeal cancer, diagnosed between 2007 and 2016, were identified. The total number of new cases treated for oral cavity and oropharynx cancer, detailed per year, are shown in the Fig. 1.

Among the different types of oral cavity cancer, tongue cancer treatment was the one with the highest number of records in the period (32.1%), while oropharyngeal cancer (C10) (48.1%) was the most frequently treated among the oropharyngeal cancer types. The number of treated cases of cancer, according to the time frame of interest and according to the primary tumor location is shown in Table 1. It is worth mentioning that 65.8% of the cases of oropharynx cancer (C10) were recorded as C10.9 (NOS – No Other Specifications).

**Table 1:** Number of cases treated for oral cavity and oropharynx cancer recorded in Brazil, 2007‒2016.

	2007	2008	2009	2010	2011	2012	2013	2014	2015	2016	Total n (%)
**Oral cavity**	4567	4811	5062	5514	5685	5436	5825	5755	5599	4545	52799
Lip	546	558	583	614	619	566	651	612	578	478	5805 (11.0)
Tongue	1421	1478	1536	1722	1796	1781	1882	1940	1831	1563	16950 (32.1)
Gums	185	186	178	222	204	209	195	230	228	184	2021 (3.8)
Floor of the mouth	695	759	879	871	942	816	942	867	872	683	8326 (15.8)
Palate	784	891	867	967	1042	1000	1015	1006	997	804	9373 (17.8)
Other unspecified parts of the mouth	936	939	1019	1118	1082	1064	1140	1100	1093	833	10324 (19.6)
**Oropharynx**	2747	2914	3253	3471	3694	3609	3748	3837	3731	3512	34516
Base of the tongue	859	970	1034	1080	1206	1109	1193	1179	1144	1092	10866 (31.5)
Tonsils (Palatine Tonsils)	593	693	648	689	704	765	741	768	744	714	7059 (20.4)
Oropharynx	1295	1251	1571	1702	1784	1735	1814	1890	1843	1706	16591 (48.1)

Regarding the sociodemographic characteristics, a large proportion of patients was male, aged 50–59 years old, predominantly Caucasians, and with a low level of schooling (incomplete Elementary education) (Table 2).

**Table 2:** Demographic characteristics of individuals treated for cancer of the oral cavity and oropharynx, Brazil, 2007‒2016.

Variables	Oral cavity (n = 52.799)		Oropharynx (n = 34.516)	
	n	%	n	%
Gender				
Male	39218	74.3	29096	84.3
Female	13581	32.7	5420	15.7
Age group^a^				
<40	2531	4.8	1146	3.3
40‒49	7728	14.6	6008	17.4
50‒59	16022	30.3	12663	36.7
60‒69	13760	26.1	9080	26.3
≥70	12750	24.2	5615	16.3
Ethnicity/skin color^b^				
Yellow	309	0.9	192	0.8
White	16658	46.9	10703	45.9
Native	47	0.1	24	0.1
Brown	16119	45.4	10619	45.5
Black	2389	6.7	1804	7.7
Level of schooling				
None/illiterate	6288	15.7	3213	12.2
Incomplete elementary school	19763	49.3	13395	51.0
Complete elementary school	7784	19.4	5377	20.5
Complete high school	4647	11.6	3176	12.1
Complete/incomplete College/University	1569	3.9	1090	4.2

^a^Variable classified as “No information” in 12 (<0.1%) of the cases.

^b^Variable classified as “No information” in 28,451 (32.6%) of the cases.

^c^Variable classified as “No information” in 21,013 (24.1%) of the cases.

**Figure 2:**
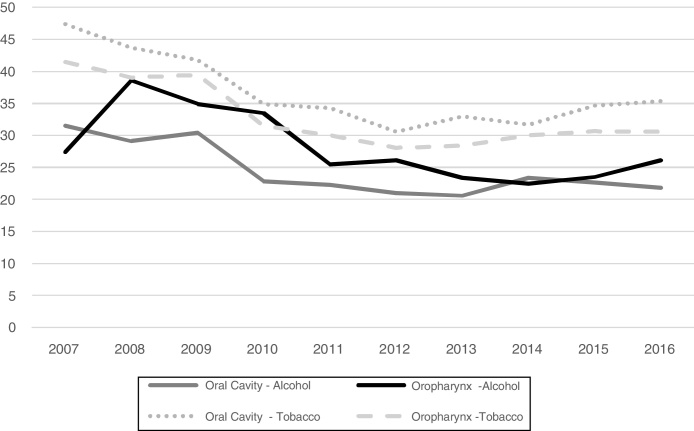
History of alcohol consumption of individuals with oral cavity (*p* =  0.05) and oropharynx (*p* = 0.03) cancer and tobacco consumption of individuals with oral cavity (*p* =  0.02) and oropharynx (*p* < 0.01) cancer per year, Brazil, 2007‒2016. ^a^Variable “alcohol consumption” classified as “No information” in 41,955 (48.0%) cases; ^b^Variable “tobacco consumption” classified as “No information” in 39,340 (45.0%) cases.

The history of alcohol or tobacco consumption over the last decade is shown in Fig. 2.

Many patients were diagnosed at an advanced stage of the disease (III or IV) (Table 3). Regarding the disease status at the end of the first treatment, a significant proportion of patients with oral cavity cancer had no evidence of the disease or were in complete remission (30.0%), whereas a considerable percentage of patients with oropharyngeal cancer died (24.8%). The first most frequently received treatment of oral cancer patients was surgery (24.0%), whereas patients with oropharyngeal cancer received chemoradiotherapy (29.2%).

**Table 3:** Clinical and care-related characteristics of individuals with cancer of the oral cavity and oropharynx, Brazil, 2007‒2016.

Variables	Oral cavity (n = 52,799)		Oropharynx (n = 34,516)	
	n	%	n	%
Staging^a^				
I/II	10751	30.6	2761	12.0
III/IV	24408	69,4	20205	88.0
Disease at the end of the first treatment^b^				
Progressing disease	2883	13,9	2145	15.9
Stable disease	4829	23,3	3333	24.8
Out of therapeutic possibility	772	3,7	536	4.0
Death	4283	20,7	3476	25.8
Partial remission	1744	8,4	1283	9.5
No evidence of disease/complete remission	6222	30,0	2676	19.9
First treatment received^c^				
Surgery	12539	24,0	2520	7.4
Surgery + CT	1174	2,2	814	2.4
Surgery + RT	4369	8,3	1132	3.3
Surgery + CT + RT	3736	7,1	2360	6.9
CT	3665	7,0	3999	11.7
CT + RT	8215	15,7	9991	29.2
RT	8383	16,0	6497	19.0
Others	1599	3,1	1284	3.8
None	8660	16,5	5603	16.4
Reason for not treating^d^				
Abandonment of treatment	564	7,3	389	6.8
Treatment complications	26	0,3	32	0.6
Advanced disease	1396	18,1	1137	20.0
Death	2004	26,0	1885	33.1
Others	2465	32,0	1574	27.6
Treatment refusal	296	3,8	154	2.7
Treatment carried out in another place	962	12,5	525	9.2

a Variable classified as “No information” in 16,729 (19.1%) of the cases and as “Not applicable” in 12,461 (14.5%) of the cases.

b Variable classified as “No information” in 39,703 (45.5%) of the cases.

c Variable classified as “No information” in 775 (0.9%) of cases.

d Variable classified as “No information” in 6,885 (7.9%) of the cases and as “Not applicable” in 67,021 (76.7%) of the cases.

## Discussion

Regarding the epidemiological, clinical and care profile of the treatment provided to patients with oral cavity and oropharyngeal cancer in Brazil, a discreet and progressive increase in the number of cases recorded in the period of interest was observed, mainly in the cases of oropharynx, which is consistent with INCA estimates.^2^ According to the Surveillance, Epidemiology and End Results Program (SEER), it was also observed that the incidence of oral and oropharyngeal cancer has increased by an average of 0.6% per year in the last decade in the United States, with the incidence of oropharyngeal and palatine tonsil cancer cases increasing on average 2.9% per year.^14^ Overall, the epidemiological profile of patients showed a similarity with the previous study on the epidemiology of head and neck cancer in Brazil, which covered the period from 2000 to 2008.^15^

The higher percentage of individuals with a low level of schooling corroborates data from a systematic review that reported, despite the lack of uniformity regarding the definition of social determinants of health among the assessed studies, that a low level of schooling, among other determinants, has a strong association with mouth cancer.^16^ Moreover, it is worth mentioning that socioeconomic determinants can influence behaviors and lifestyles, particularly those related to cancer risk factors, such as smoking, alcoholism and poor diet.^17^

The fact that most cases continue to be diagnosed at advanced clinical stages is a matter of concern, which may compromise the rate of cure and/or survival of these patients. Considering that this finding corresponds to that of almost two decades ago, it is believed that there may have been difficulties regarding the promotion of preventive measures/strategies and early diagnosis.^15^ This situation can result in higher costs for the healthcare system, since most T3 and T4 tumors require multimodal therapy. Moreover, the survival rate and quality of life of these patients is generally much lower than that of patients diagnosed at early stages.^9^

The late diagnosis reinforces the need for actions aimed at educating the population and the need for training primary healthcare professionals to detect malignant lesions at earlier stages. Screening for cancer of the lip and oral cavity, for instance, is not recommended as an early detection strategy; however, the training of healthcare professionals, particularly of dental surgeons in the basic health units, would be an appropriate strategy to address this population demand.^2^, ^18^

When analyzing the trend of the history of consumption of alcoholic beverages and tobacco products over the past few years, a reduction is observed, mainly in relation to tobacco consumption. A case-control study reported that the HNC fractions attributable to smoking were more significant than for alcohol consumption.^17^ Data from the INHANCE study show that, compared to non-smokers, any number of cigarettes increases the risk of HNC (0–3 cigarettes per day: OR = 1.52; 95%CI, 1.21–1.90) and the consumption of 5–10 cigarettes a day more than doubles the risk of developing HNC (overall OR = 2.6; 95%CI, 2.00–3.40).^19^

According to Wünsch Filho et al., the reduction of smoking among the Brazilian population may have an influence on the reduction of morbidity and mortality due to HNC in the future.^20^ The prevalence of alcohol consumption, on the other hand, still shows an upward trend.^21^ In agreement with these results, the data presented in this work, which apparently indicate a trend toward a reduction in the history of alcohol and tobacco use, do not necessarily represent a counterpoint to the indicators described in the literature. An important factor that has an impact on the picture of consumption of these risk substances in the country is related to the characteristics of the Brazilian hospital cancer record itself, whereas some fields (such as variables related to alcoholism and smoking) do not require the compulsory filling out on the screen of the SisRHC.

Thus, the main limitation of this study is related to the use of secondary data from an extremely important information system, but not free of flaws regarding incomplete files related to the variables of interest (missing information). Despite the development of cancer registries over the years, another important challenge is their improvement, making them able to provide complete data on epidemiology and allowing a more accurate analysis of the behavior of cancer cases in Brazil. The fact that most cases of the oropharynx (C10) are registered as C10.9 (NOS – No Other Specifications), for instance, should encourage the need to better classify the detailed primary location of this type of cancer in the several cancer care services.

Moreover, relevant etiological data on HNC, such as HPV status, are not available. There is a case-control study in the literature that demonstrated an association between HPV infection and the risk of HNC, regardless of the use of tobacco and alcohol.^9^ Still, it is known that HPV-positive patients respond better to chemoradiotherapy. These cases, however, are still rare in Brazil.

It is noteworthy that the SisRHC data do not represent the totality of new cancer cases diagnosed in the country, but the distribution of treated/registered cases. Therefore, it is not possible to calculate, based on these records, the estimates and projections for measures of frequency of oral cavity and oropharynx cancers, as it is done in population-based studies.

The approach of descriptive epidemiology addressed in this study allowed the discussion of some data regarding the epidemiology of oral cavity and oropharynx cancer in Brazil from 2007 to 2016. Descriptive epidemiology is crucial to identify the upward trends regarding the incidence rates and the distributions according to personal attributes, allowing the characterization of the disease behavior, showing its changes over time, and indicating new control strategies. In this sense, the present study contributes to define public health policies, aiming to guide better care for such complex patients.

## Conclusion

Taken together, the results of the clinical, epidemiological and care characterization make it possible to conclude that during the period of interest there was a discrete and progressive increase in the distribution of new cases of the assessed cancer types, an almost stationary and regular distribution of care to such cancer patients, as well as a trend towards a reduction in records regarding the history of consumption of alcohol and tobacco.

Although it does not represent variations in the incidence and prevalence of oral cavity and oropharyngeal cancers in the Brazilian population, the relative increase in the number of consultations for such causes in the studied decade can be understood as an important indicator of demand for high-technological density services and a partial picture of the established capacity for providing cancer care in the country.

The predominantly late diagnosis and the associated low level of schooling of the assessed patients show the importance of improving the strategic actions of health education and primary prevention, aiming to promote the early diagnosis of malignant neoplasms of the oral cavity and oropharynx.

## Sources of funding

This study was carried out with support from *Coordenação de Aperfeiçoamento de Pessoal de Nível Superior* ‒ Brazil (CAPES) ‒ Funding Code 001.

## Conflicts of interest

The authors declare no conflicts of interest.
